# Brain activation to high-calorie food images in healthy normal weight and obese children: a fMRI study

**DOI:** 10.1186/s40608-018-0209-1

**Published:** 2018-12-03

**Authors:** Amjad Samara, Xuehua Li, R. T. Pivik, Thomas M. Badger, Xiawei Ou

**Affiliations:** 1 Arkansas Children’s Nutrition Center, Little Rock, AR USA; 2 Department of Pediatrics, University of Arkansas for Medical Sciences, Little Rock, AR USA; 3 Department of Radiology, University of Arkansas for Medical Sciences, Little Rock, AR USA; 4 Arkansas Children’s Research Institute, Little Rock, AR USA

**Keywords:** fMRI, Children, Obesity, High-calorie food, Dorsomedial prefrontal cortex, Parahippocampal gyrus

## Abstract

**Background:**

Understanding how normal weight and obese young children process high-calorie food stimuli may provide information relevant to the neurobiology of eating behavior contributing to childhood obesity. In this study, we used fMRI to evaluate whether brain activation to high-calorie food images differs between normal weight and obese young children.

**Methods:**

Brain activation maps in response to high-calorie food images and non-food images for 22 healthy, 8–10-years-old children (*N* = 11/11 for normal weight/obese respectively) were generated and compared between groups.

**Results:**

When comparing brain activation differences in response to viewing high-calorie food versus non-food images between normal weight and obese children, group differences were observed in areas related to memory and cognitive control. Specifically, normal weight children showed higher activation of posterior parahippocampal gyri (PPHG) and dorsomedial prefrontal cortex (DMPFC). Further ROI analyses indicated higher activation strength (Z scores) in the right PPHG (*p* = 0.01) and higher activation strength (*p* < 0.001) as well as a larger activation area (*p* = 0.02) in the DMPFC in normal weight than obese children.

**Conclusions:**

Normal weight and obese children process high-calorie food stimuli differently even from a young age. Normal weight children exhibit increased brain activation in regions associated with memory and cognitive control when viewing high-calorie food images.

**Electronic supplementary material:**

The online version of this article (10.1186/s40608-018-0209-1) contains supplementary material, which is available to authorized users.

## Background

Childhood obesity is a significant public health concern and its prevalence has continued to increase in the last decades [1]. In 2011–2014, the rate of obesity among children and adolescents between 2 and 19 years of age in the United States was 17.0% and extreme obesity was 5.8% [1]. Overweight and obese children are at increased risk of being overweight or obese as adults and for the adverse health consequences associated with these conditions [2]. For example, childhood obesity is strongly linked to metabolic complications and chronic illnesses like hypertension, diabetes mellitus, and cardiovascular diseases [3], and cognitive function and academic performance may also be affected [4]. Childhood obesity is related to some risk factors such as genetic, physical activity, and family environment [5], but unhealthy eating habits may also play an important role [6]. New insights about the brain-obesity connection and the neurobiology of eating behavior in young children would guide our understanding of the behavioral correlates of childhood obesity. This information may inform the development of earlier and more effective behavioral interventions to address childhood obesity.

Recently, fMRI has been extensively used to study brain activation in response to food stimuli. When stimulated by food images, specific brain regions may be activated, including orbitofrontal cortex, insula, striatum, and amygdala [7–10]. Factors influencing the specific activation pattern observed include the motivational status of participants, which reflects how hungry they are [7], and their perception of the energy density of food images, i.e. high or low-calorie content [11]. Several studies compared brain activation to food stimuli in obese and normal-weight adults and have reported group differences in response patterns; specifically increased activation in reward-related brain regions in obese adults [12]. Meanwhile, brain structure and function continue to develop during childhood and different patterns of brain activation in children in response to food stimuli are expected [13]. This limits the generalizability of adult’s studies to young children and necessitates additional studies for children. However, few previous studies have compared brain activation in response to food stimuli in obese and normal weight children. For example, Davids et al. reported that obese children exhibit increased activation in the dorsolateral prefrontal cortex in response to food images, whereas, normal weight children exhibit higher activation in the caudate and the hippocampus [14]. Few other studies investigated the effect of food energy density on brain activation patterns in normal children. For example, Killgore et al. reported differences in brain activation in response to high and low-calorie food images in normal children and suggested that these differences could be related to cognitive development and inhibitory control [15]. Nevertheless, brain activation in response to high-calorie food stimuli and how it relates to body weight in children have not been fully characterized. Further characterization of brain activation in response to high-calorie food stimuli in different motivational status i.e. hunger and satiety in children are still necessary. A couple of studies reported brain activation differences in response to images of high-energy-density or unhealthy food compared with low-energy-density or healthy food and related the findings to measures of child body composition [13, 16]. In the first study, Fearnbach et al. reported higher activation in the left thalamus in response to high-energy-density food relative to low-energy-density food; and a significant positive association between activation for the contrast of high-energy-density vs. low-energy-density food in the right substantia nigra, and child fat-free mass [16]. In the second study, lower activation in the bilateral dorsolateral prefrontal cortex in response to unhealthy compared with healthy food was associated with higher body mass index in 10–12-year-old children [13]. Developmental changes in functional brain responses to food images indicated that activation patterns observed in these studies may differ in younger children [15]. Moreover, exploring whether differences in brain activation to food stimuli would exist in younger children may provide a time window for earlier interventions to prevent childhood obesity.

In this study, we used fMRI to evaluate brain activation differences to high-calorie food images in preadolescent normal weight and obese children aged 8–10 years old. We hypothesize that, while viewing high-calorie food images, normal children will exhibit higher activation in brain regions associated with cognitive control when compared with obese children.

## Methods

### Study population

Healthy normal weight (BMI < 75th percentile) and obese (BMI > 95th percentile) children (age 8–10 years) were recruited for this study. All experimental procedures were approved by the institutional review board at the University of Arkansas for Medical Sciences and informed consents/assents were obtained from all children’s parents and children participating in the study. **Inclusion criteria:** parental reports of full-term gestation and birth weight between 5th – 95th percentiles; parental report of right-hand dominance; and parental report of no obesity related (such as diabetes) or other medical conditions. **Exclusion criteria:** maternal diabetes; maternal alcohol, tobacco, or drug use during pregnancy; chronic sleep disorder; history of psychological or psychiatric diagnoses; history of neurological impairment or injury; surgical implant or other foreign object in the body; dental work which may cause artifacts in MRI; known claustrophobia; and high likelihood of inability to tolerate loud noise from the MRI scanner. Twenty-seven children were initially enrolled; 1 voluntarily withdrew; 2 did not attempt the MRI; 1 did not complete the fMRI, and 1 had an invalid fMRI scan due to excessive motion. In total, 22 children (11/11 for normal weight/obese) completed the scan and had valid fMRI as well as structural imaging data and were included in this study. The demographic information for all subjects is listed in Table 1.

**Table 1:** Demographic information for the participants

	Normal weight (*N* = 11)	Obese (*N* = 11)	*p* value
Sex (male/female)	5/6	6/5	1
Age at MRI (years)	9.77 ± 0.70	9.11 ± 0.91	0.10
BMI	15.85 ± 1.07	24.74 ± 3.37	< 0.001
BMI percentile	11th–57th	95th–99th	

#### MRI/fMRI data acquisition

**Fig. 1:**
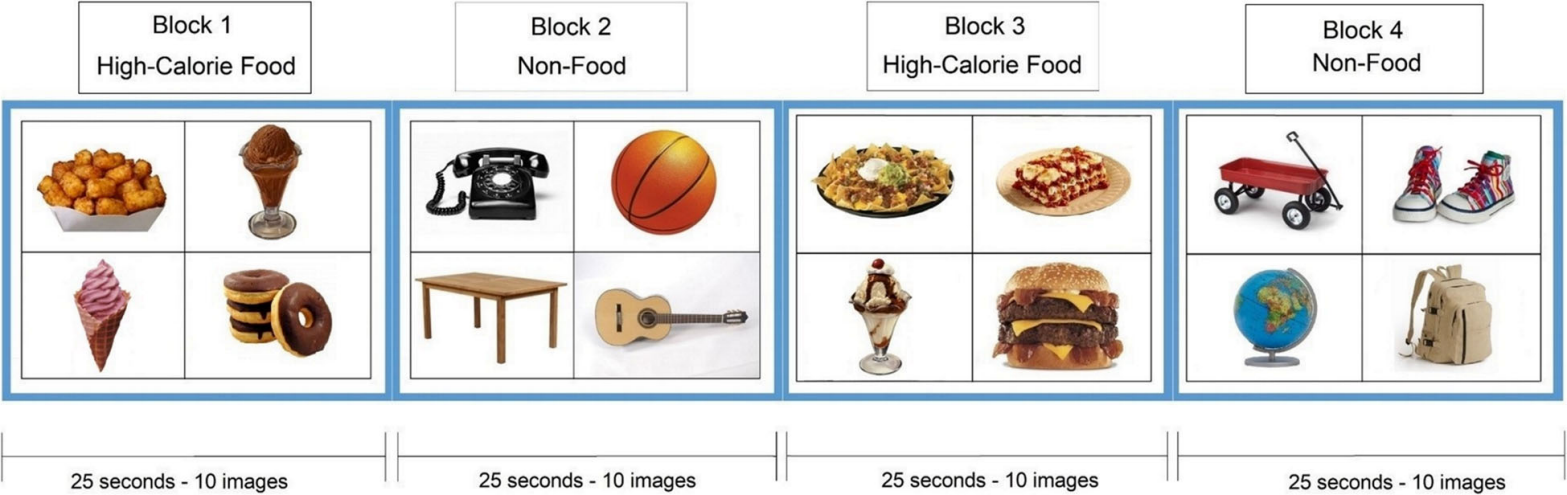
A four block example of high-calorie food images and non-food images used in fMRI paradigm

All participants had an MRI/fMRI examination at the Arkansas Children’s Hospital on a 1.5 Tesla Achieva MRI scanner (Philips Healthcare, Best, The Netherlands) with an 8-channel SENSE head coil. All scans were done on Saturday mornings at around 9 am. All participants consumed breakfast before scans and the fMRI studies were performed at approximately the same morning time of the day, which made confounding factors, such as motivational status, less relevant between groups. Imaging sequences included a sagittal T1-weighted 3D turbo field echo sequence for structural MRI, with 7.4 ms TR, 3.5 ms TE, 8° flip angle, no slice gap, 1 mm × 1 mm × 1 mm acquisition voxel size, 256 × 232 × 150 matrix size; and an axial single-shot gradient echo EPI sequence for fMRI, with 2500 ms TR, 50 ms TE, 2.4 mm × 2.4 mm × 5.0 mm acquisition voxel size, 128 × 128 reconstruction matrix size, 20 slices, 4 dummy scans, and 120 dynamics. An Eloquence fMRI system (Invivo Corporation, Orlando, FL, US) was used to display the fMRI paradigm (and play a movie during the structural MRI scan) and was synchronized with the MRI scanner. The fMRI paradigm included a picture viewing task, in which a single image of a high-calorie food or non-food item was displayed on the center of the screen for each trial and participants were instructed to look at the screen all the time during the task. A block design consisting of 6 non-food blocks (which served as baseline condition) and six food blocks (which served as activation condition) alternating with each other was used for the fMRI. Each block lasted 25 s and included ten trials with each trial displaying one image on the screen for 2.5 s. Therefore, there were ten images for each block, and 60 non-food and 60 high-calorie food images in total for the whole fMRI paradigm. The non-food and high-calorie food images were all different, balanced for size and contrast, and were all common items with high recognition and familiarity to the participants. The high-calorie food was defined as energy density ≥ 1.5 kcal/gram. The mean energy density of food presented in the images is 3.2 kcal/gram. A full list of these high-calorie food images and non-food items is included in the Additional file 1. An example of fMRI paradigm is shown in Fig. 1.

#### MRI/fMRI data analysis

All MRI data were exported to a workstation with FMRIB Software Library V5.0 (FSL, created by the Functional MRI of the Brain Analysis Group, University of Oxford, UK) installed on a VMware Linux virtual machine (VMware, Inc., Palo Alto, CA USA). The FSL’s FEAT program was used for the fMRI data analysis. Specifically, all fMRI images were preprocessed for motion detection and correction using FSL commands such as fsl_motion_outliers and fsl_regfilt, and maximum rotation/translation were limited to 2.5°/1.5 mm, respectively. Additional preprocessing tools in FEAT were used, including MCFLIRT for motion correction, BET for non-brain tissue removal, slice time correction, high pass temporal filtering, and spatial smoothing using a Gaussian kernel with a 5 mm FWHM. By using both linear and non-linear registration programs (FLIRT/FNIRT), the preprocessed fMRI images were registered to the T1-weighted 3D structural images for each subject and then normalized to a customized template that was created in FSL using the T1 3D images for all subjects. Time series statistical analysis was performed using the FMRIB Improved Linear Model. Standard and extended motion parameters (as estimated by the MCFLIRT) were included as confounding explanatory variables in the model. Results were entered into the higher level analysis of the FEAT program to compute for average activation maps for each group. Independent region of interest (ROI) method was used to compare the brain activation in the normal weight and obese groups. Specifically, anatomical regions that indicated apparent activation differences on the average activation maps in FSL were sketched in a separated software MATLAB (The MathWorks, Inc., MA, USA) as the ROIs, with the sketching of ROI based solely on the anatomy of the full region as shown on the T1 weighted high resolution images to ensure the construction of the ROI is independent of the activation maps, and then the mean Z score (activation strength) as well as activated imaging voxels in each ROI for each subject were calculated and compared between groups.

### Statistics

For comparisons of demographic parameters and ROI analysis of fMRI activation results (including mean Z scores and numbers of activated imaging voxels) between normal weight and obese children, non-parametric Wilcoxon rank-sum tests were used for numerical parameters, while Fisher’s Exact Test was used for categorical variables. *p* < 0.05 was regarded as significant. For the calculation of average activation maps for each group in the FEAT program, statistical significance was defined as a threshold of Z score > 2.3 and *p* < 0.05 (whole brain cluster-wise corrected).

## Results

**Fig. 2:**
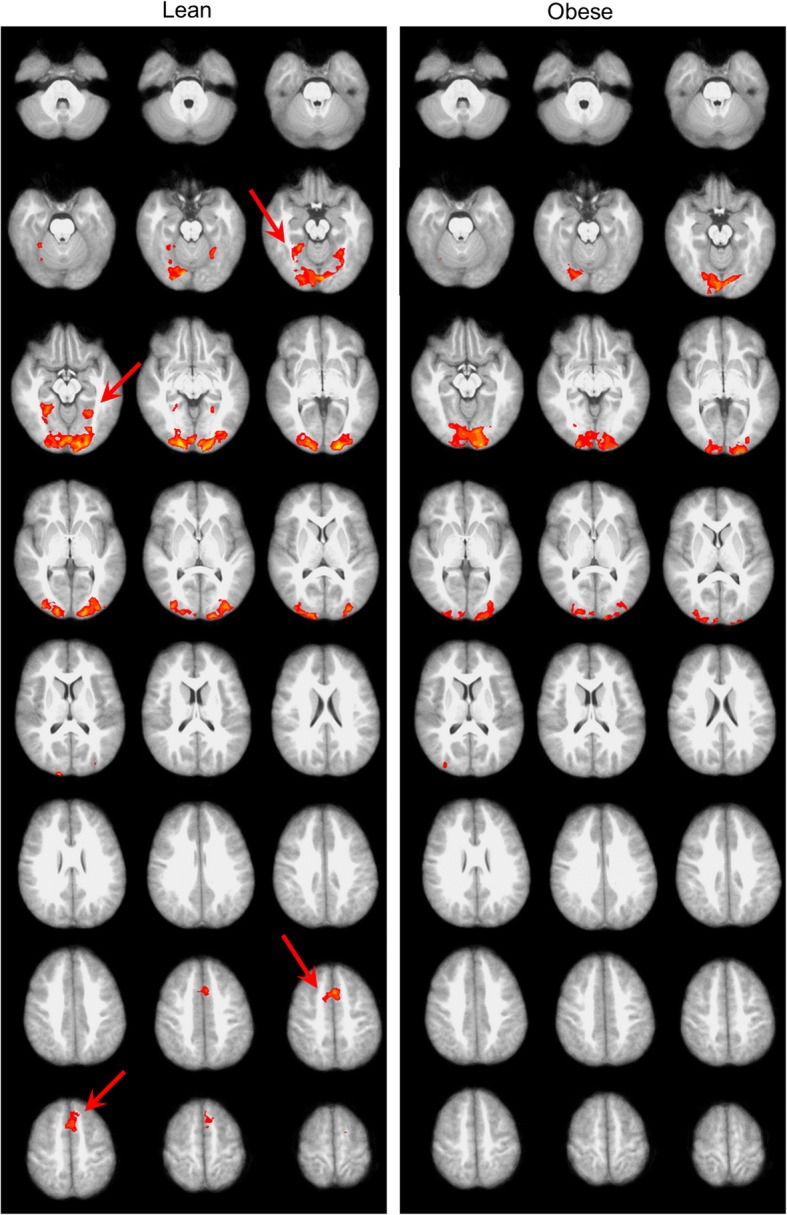
Mean activation maps for the normal weight and obese children (at a Z score threshold of 2.3 and *p* < 0.05, corrected for the voxel-wise multiple comparisons). Red arrows point to regions (PPHG and MPFC) that were significantly activated in the normal weight but not obese children

The normal weight and obese children groups did not differ in sex composition (*p* = 1), or age at MRI (*p* = 0.10), but as per experimental design were significantly different in BMI (*p* < 0.001). The picture viewing task with non-food images as baseline condition and high-calorie food images as activation condition consistently activated the visual cortex for both normal weight and obese children (Fig. 2). However, both left and right posterior parahippocampal gyri (PPHG) were activated by food images for the normal weight but not the obese children (Fig. 2). Likewise, the dorsomedial prefrontal cortex (DMPFC) was activated for the normal weight but not the obese children (Fig. 2).

**Fig. 3:**
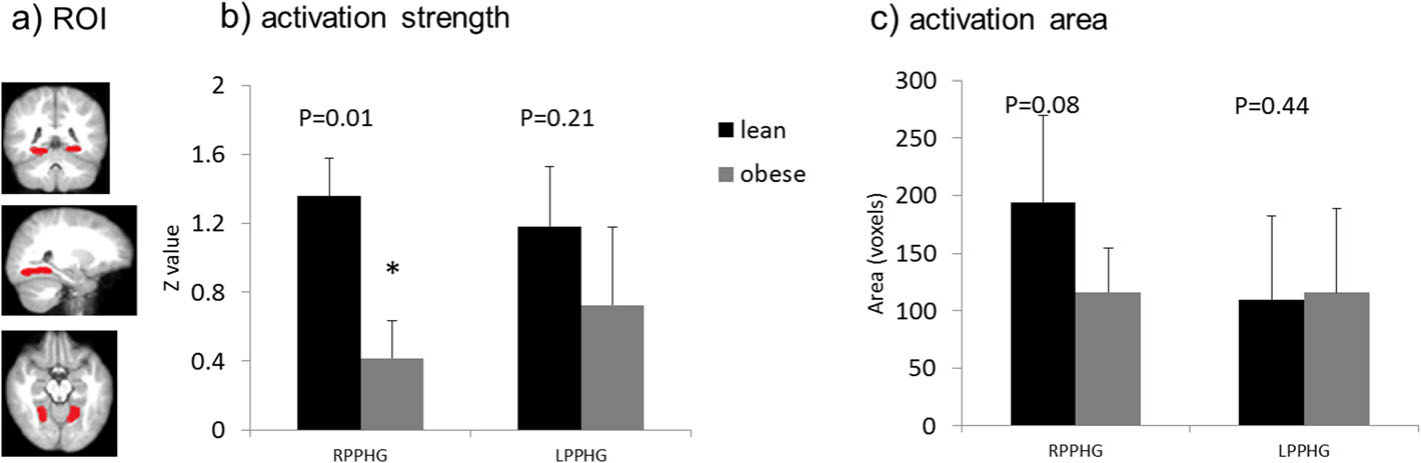
Group comparison of activation strength and the total activated area in the PPHG. **a**) illustration of the region-of-interest (ROI) selection; **b**) group comparison of activation strength (mean z value) in left and right PPHG; and **c**) group comparison of activation area (number of voxels activated) in left and right PPHG. * *p* < 0.05 for the non-parametric Wilcoxon rank-sum test

**Fig. 4:**
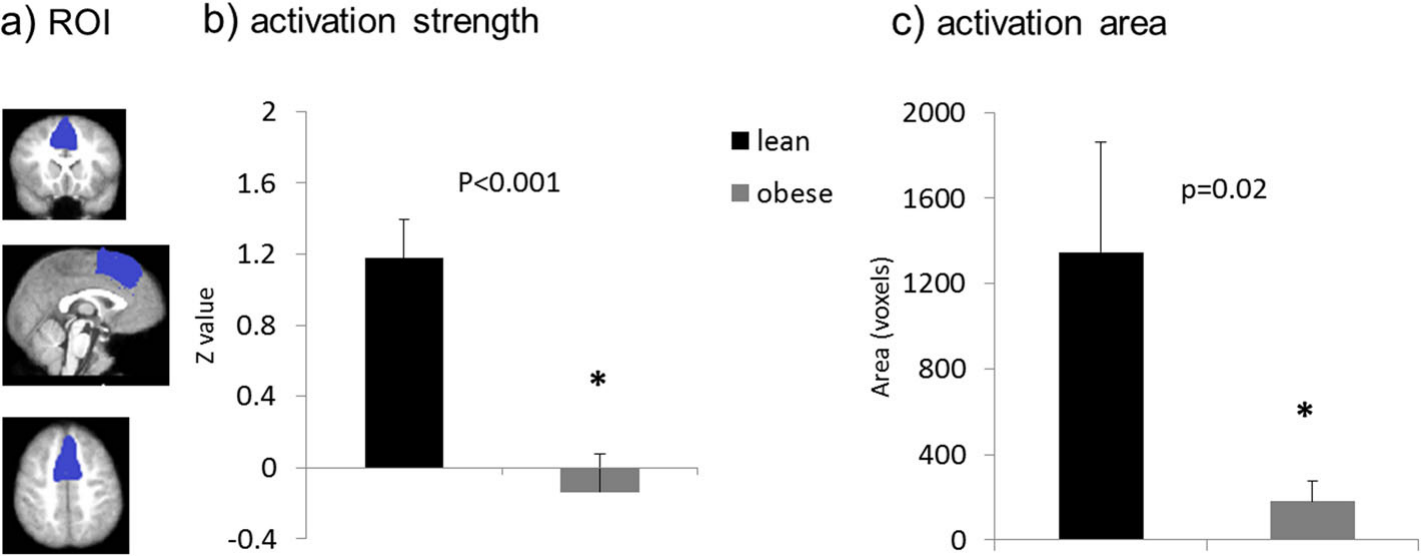
Group comparison of activation strength and the total activated area in the DMPFC. **a**) illustration of the ROI selection; **b**) group comparison of activation strength (mean z value) in DMPFC; and **c**) group comparison of activation area (number of voxels activated) in DMPFC. * *p* < 0.05 for the non-parametric Wilcoxon rank-sum test

The Left and right PPHG (Fig. 3a), as well as the DMPFC (Fig. 4a), were sketched based on anatomy using the customized T1-weighted imaging template as the ROIs for further post hoc analysis of the activation maps. These analyses revealed that in the right PPHG (RPPHG), normal weight children had significantly stronger (*p* = 0.01) mean activation strength (as reflected by Z scores) and tended to have a larger mean activation area (*p* = 0.08) than obese children; in the left PPHG (LPPHG), there was a trend for stronger mean activation strength in normal weight than obese children, but both groups showed comparable mean activation areas (Fig. 3b, c). In the DMPFC, normal weight children had both significantly stronger mean activation strength (*p* < 0.001) and larger mean activation area (*p* = 0.02) compared with obese children (Fig. 4b, c).

## Discussion

Our results showed significant differences in brain activation patterns to high-calorie food versus non-food images between healthy normal weight and obese children. Specifically, the PPHG and the DMPFC were activated in normal weight but not in obese children based on the mean activation maps; additional ROI analysis showed significant differences in mean activation strength of the right PPHG, and in both mean activation strength and mean activation area of the DMPFC. Brain activation differences in response to food images between the normal weight and obese preadolescent children in our study are not surprising, as differences were previously documented in other age groups. For example, several studies reported significant differences in activation in striato-limbic regions including putamen/caudate, insula, orbitofrontal cortex and amygdala in obese versus normal-weight adults [12]. Moreover, these differences are also reported in normal weight versus obese children when viewing food images [14, 17]. It is noteworthy that brain activation patterns in response to food stimuli can be affected by the motivational status of participants and energy density of food [7, 11]. For example, hunger and satiety rates in adults were positively correlated with activation in orbitofrontal cortex and insula [9, 18]. In other adult studies, high-calorie food increased activation in satiety-related regions like lateral orbitofrontal cortex, and low-calorie food increased activation in hunger-related regions like medial orbitofrontal and insular cortex [12, 19].

In our study, visual cortex showed increased activation in both normal weight and obese children when viewing high-calorie food versus non-food images. Activation of the occipital region is a common finding in viewing food images [10, 11]. In a meta-analysis conducted by Van der Laan et al. [11], lateral occipital complex (LOC, a component of visual association cortex) was one of the most commonly activated regions in viewing food vs. non-food images in adults. It was suggested that activation of LOC cannot be explained by its role in object recognition since food and non-food images were usually matched to neutralize this effect. A more likely explanation is that increased activation in LOC is related with higher alertness to food versus non-food objects and, as a result, stronger visual cortex activation [11]. No significant difference in activation of visual cortex was noticed in our study when comparing normal weight with obese children.

The PPHG was significantly more activated in normal weight versus obese children in response to high-calorie food images. It is widely believed that hippocampal and parahippocampal networks are related to declarative memory functions. Parahippocampal gyrus plays a crucial role in memory encoding and retrieval [20]. Similar to our study, Davids et al. found increased activation in the hippocampus and the parahippocampal gyrus when comparing normal weight versus obese children viewing food images [14]. It was suggested that this pattern of activation could also indicate increased alertness for food images. In the same study [14], normal weight children also showed increased activation in other areas such as anterior cingulate cortex, fusiform gyrus, thalamus, caudate and parts of the visual cortex. These differences were not observed in our study, presumably due to different paradigm designs that two groups of food images (neutral and pleasant) were used in their study. Another study showed that increased parahippocampal gyrus volume in children is associated with a lower increase in BMI [21], suggesting a role of parahippocampal gyrus and its functioning associated with childhood obesity.

The second brain region that was significantly more activated in normal weight versus obese children in our study was the DMPFC. Several neuroimaging studies in adults have linked DMPFC activation to social cognition, mentation, and processing information related to social judgment [22]. DMPFC also plays an important role in decision making and response control in a variety of contexts [23, 24], particularly under conflicting and uncertain conditions [25, 26]. In adults, value signal of different options is encoded in MPFC and is also activated when making food decisions [27, 28]. Van Meer et al. suggested that children tend to make their food choices based on taste rather than healthiness [29]. Because of the higher activation in memory-related brain regions in healthy children viewing high-calorie food images in our study, we postulate that recall of previous knowledge about healthiness of food presented in the images in healthy children may have had an effect in our study. This knowledge might have come from their common knowledge received from parent or school education. Besides, higher DMPFC activation in normal weight children when viewing high-calorie food images may indicate increased conflict and more information processing regarding the value of food images. This higher DMPFC activation in healthy children could also be associated with different attitudes toward food and, together with activation in the parahippocampal gyrus, retrieval of associations with different past experiences related to food choices.

The food addiction model of obesity links obesity to abnormal activation in cognitive control and some motivation-reward pathways in the brain [30]. Nevertheless, this model of obesity is still controversial [31]. The absence of activation differences in many important motivation-reward areas in our study suggests that responses to high-calorie food images may not be explained merely by the food addiction model of obesity. The differences we observed were mostly in cognitive control and memory areas rather than reward circuits as classically depicted in adult studies. This could suggest how normal weight and obese children differently recall past understanding related to food healthiness and react to food stimuli. Due to the lack of assessment of children’s attitudes towards the presented food images, we could not show enough evidence to support an assumption that children’s knowledge about healthy food explains the differences in brain activation patterns. Nevertheless, our assumption that brain activation differences are related to instant recall of presumed previous education about food healthiness could still accurately point toward the importance of early and sustained education in children about healthy food choices. Further research would be necessary to confirm these relationships.

There are some limitations to this study. First, there were no food-related behavioral assessments to correlate with our neuroimaging findings, e.g., rating from participants regarding the degree of “like” or “want” or awareness of “healthy” or “unhealthy” for the food in the images. Second, breakfasts were not standardized and we relied on parental and children’s report to confirm breakfast intake and similar timing. Lastly, although the brain activation differences we observed were significant between groups, the sample size in our study was relatively small. This could have also limited our ability to detect brain activation differences in other brain regions. Nevertheless, the results of our study provided further evidence that normal-weight children manifest different neuronal activation patterns in processing images of high-calorie food when compared with obese children, even at a young age.

## Conclusions

In summary, functional MRI brain activation patterns to high-calorie food versus non-food images differ between normal-weight and obese children (age 8–10 years). The observed group differences in memory and cognitive control brain regions indicate different responses toward high-calorie food stimuli between normal-weight and obese young children and point toward the importance of early and sustained education in children about healthy food choices. The findings of our study add to our understanding of brain-obesity association in childhood obesity.

## Additional file

Additional file 1: A list of high-calorie food and non-food items used in fMRI paradigm. (DOCX 15 kb)

## Abbreviations

BMI: body mass index
DMPFC: dorsomedial prefrontal cortex
EPI: echo-planer imaging
FMRI: functional magnetic resonance imaging
FSL: FMRIB Software Library
FWHM: full width at half maximum
LOC: lateral occipital complex
PPHG: posterior parahippocampal gyri
ROI: region of interest
TE: echo time
TR: relaxation time
