# Supplementary material for: Brain activation to high-calorie food images in healthy normal weight and obese children: a fMRI study
Source: BMC Obes. 2018 Dec 3;5:31. doi: 10.1186/s40608-018-0209-1 (PMC6276149; doi:10.1186/s40608-018-0209-1)
Supplement: Supplementary file 1 — A list of high-calorie food and non-food items used in fMRI paradigm. (DOCX 15 kb) [file 40608_2018_209_MOESM1_ESM.docx]

**Additional file 1 - A List of high-calorie food and non-food items used in fMRI paradigm:**

**High-Calorie food:**

1. Ice cream (image1)
2. Ice cream (image2)
3. Ice cream (image3)
4. Ice cream (image4)
5. Jelly
6. Lasagna (image1)
7. Lasagna (image2)
8. Macaroni cheese
9. Muffin
10. Nachos
11. Beef nachos
12. Spaghetti
13. Pastry
14. Pizza (image1)
15. Pizza (image2)
16. Pizza (image3)
17. Chocolate popsicle
18. Red cake
19. Full rack of beef ribs
20. Cinnamon roll
21. Root beer float
22. Croissant sandwich
23. Nougat Candy
24. Snow cone
25. Steak
26. Sugar cookie
27. Vanilla Sundae Ice Cream
28. Tater tots (image1)
29. Tater tots (image2)
30. Waffles
31. Twinkie
32. Apple pie
33. Bacon
34. Doughnut holes
35. Barbecue ribs
36. Jelly beans
37. Brownies
38. Burger
39. Cake (image1)
40. Cake (image2)
41. Candy chocolate
42. Cheese
43. Cheese cake
44. Cheese stick
45. Rocky road ice cream
46. Chicken alfredo
47. Fried chicken
48. Banana chips
49. Chocolate bar
50. Chocolate pie
51. Chocolate chip cookie
52. Corn dog
53. Cupcake (image1)
54. Cupcake (image2)
55. Doughnut (image1)
56. Doughnut (image2)
57. Potato French fries
58. Gummy bears
59. Hot chocolate
60. Hotdog

**Non-food items:**

1. Alarm
2. Backpack
3. Bag
4. Balloon
5. Baseball
6. Basketball
7. Bed
8. Bell
9. Belt
10. Birdhouse
11. Blanket
12. Jeans
13. Board
14. Bow
15. Bucket
16. Bus
17. Chainsaw
18. Chair
19. Couch
20. Dice
21. Flashlight
22. Slipper
23. Glasses
24. Globe
25. Glove
26. Goggle
27. Guitar
28. Hammer
29. Helmet
30. Jacket
31. Key
32. Ladder
33. Lamp
34. Lawn mower
35. Leaf
36. Pencil
37. Phone
38. Pom-pom
39. Pumpkin
40. Lawn rake
41. Scissor
42. Shoes
43. Shovel
44. Sleep
45. Socks
46. Star
47. Bar stool
48. Sunglass
49. Table
50. Tape
51. Tennis ball
52. Tent
53. Tree
54. Trumpet
55. Tuba
56. Umbrella
57. Violin
58. Wago
59. Wheel
60. Yarn
